# Supplementary material for: Improving the oxygen redox reversibility of Li-rich battery cathode materials via Coulombic repulsive interactions strategy
Source: Nat Commun. 2022 Mar 2;13:1123. doi: 10.1038/s41467-022-28793-9 (PMC8891320; doi:10.1038/s41467-022-28793-9)
Supplement: Supplementary file 1 — Supplementary Information [file 41467_2022_28793_MOESM1_ESM.pdf]

Supplementary Information

Improving the oxygen redox reversibility of Li-rich battery cathode materials via Coulombic repulsive interactions strategy

Qingyuan Li[1], De Ning[2,3], Deniz Wong[2], Ke An[4], Yuxin Tang[5], Dong Zhou[2], Götz Schuck[2], Zhenhua Chen[6], Nian Zhang[7], Xiangfeng Liu[1,8*]

[1]Centre of Materials Science and Optoelectronics Engineering, College of Materials Science and Optoelectronic Technology, University of Chinese Academy of Sciences, Beijing 100049, P. R. China

[2]Department of Dynamics and Transport in Quantum Materials and Department of Structure and Dynamics of Energy Materials, Helmholtz-Zentrum Berlin für Materialien und Energie, Hahn-Meitner-Platz 1, Berlin 14109, Germany

[3]Centre for Photonics Information and Energy Materials, Shenzhen Institutes of Advanced Technology, Chinese Academy of Sciences, Shenzhen 518055, P.R. China

[4]Neutron Scattering Division, Oak Ridge National Laboratory, Oak Ridge, Tennessee, 37830, USA

[5]College of Chemical Engineering, Fuzhou University, Fuzhou 350116, P. R. China

[6]Shanghai Synchrotron Radiation Facility, Shanghai Institute of Applied Physics, Chinese Academy of Sciences, Shanghai 201204, P. R. China

[7]Shanghai Institute of Microsystem and Information Technology, Chinese Academy of Sciences, Shanghai, 200050, P. R. China.

[8]CAS Centre for Excellence in Topological Quantum Computation, University of Chinese Academy of Sciences, Beijing 100190, China

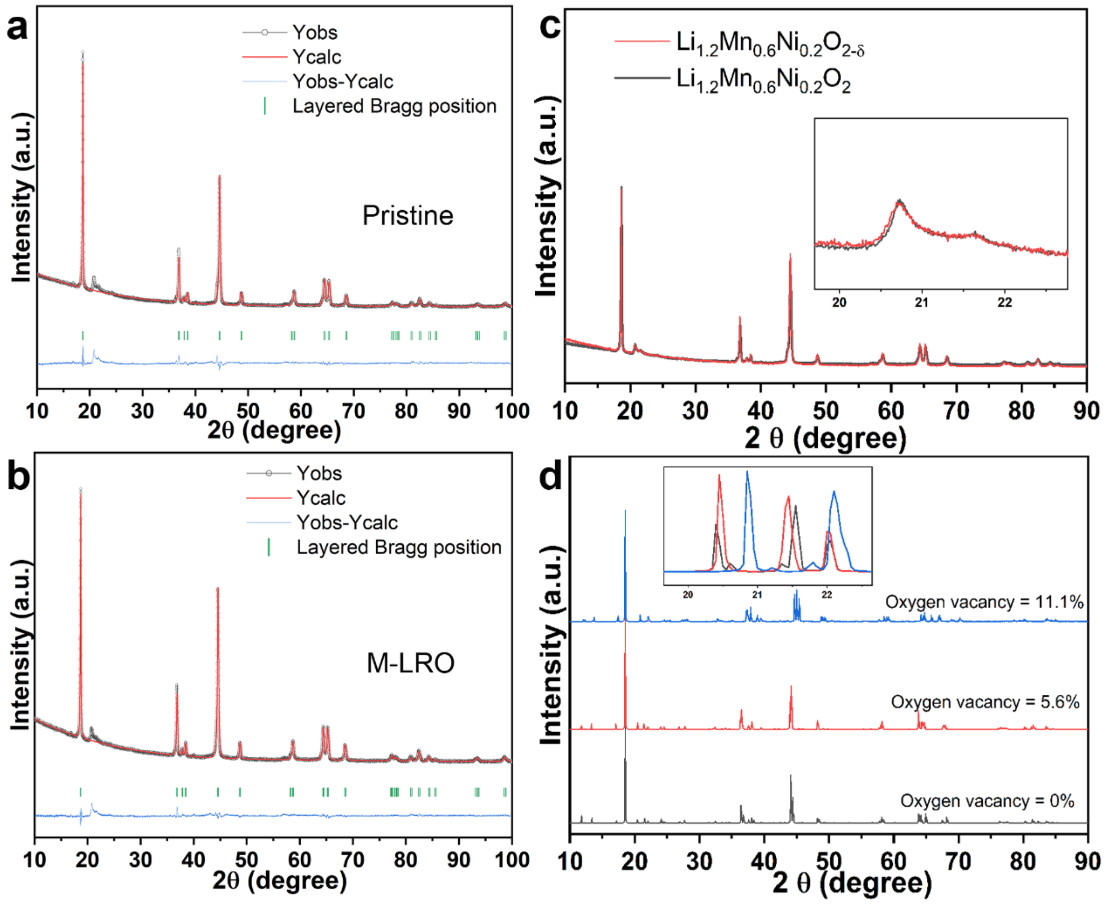

Supplementary Figure 1. (a) Rietveld refinement of the pristine ($Li_{1.2}Mn_{0.6}Ni_{0.2}O_2$) and (b) M-LRO ($Li_{1.2}Mn_{0.6}Ni_{0.2}O_{2-\delta}$) XRD patterns. (c) Normalized XRD pattern of both samples, and the inset shows the superlattice peaks. (d) Simulated XRD diffraction peaks of different oxygen vacancies. It can be seen from (c) that 5% oxygen vacancies and reduced manganese have almost no effect on the superlattice peak, which is consistent with the simulation results. However, when the concentration of oxygen vacancies reaches 11.1%, the diffraction peaks move significantly, which may come from the destruction of the structure by the excessive number of oxygen vacancies. The term a.u. means arbitrary units.

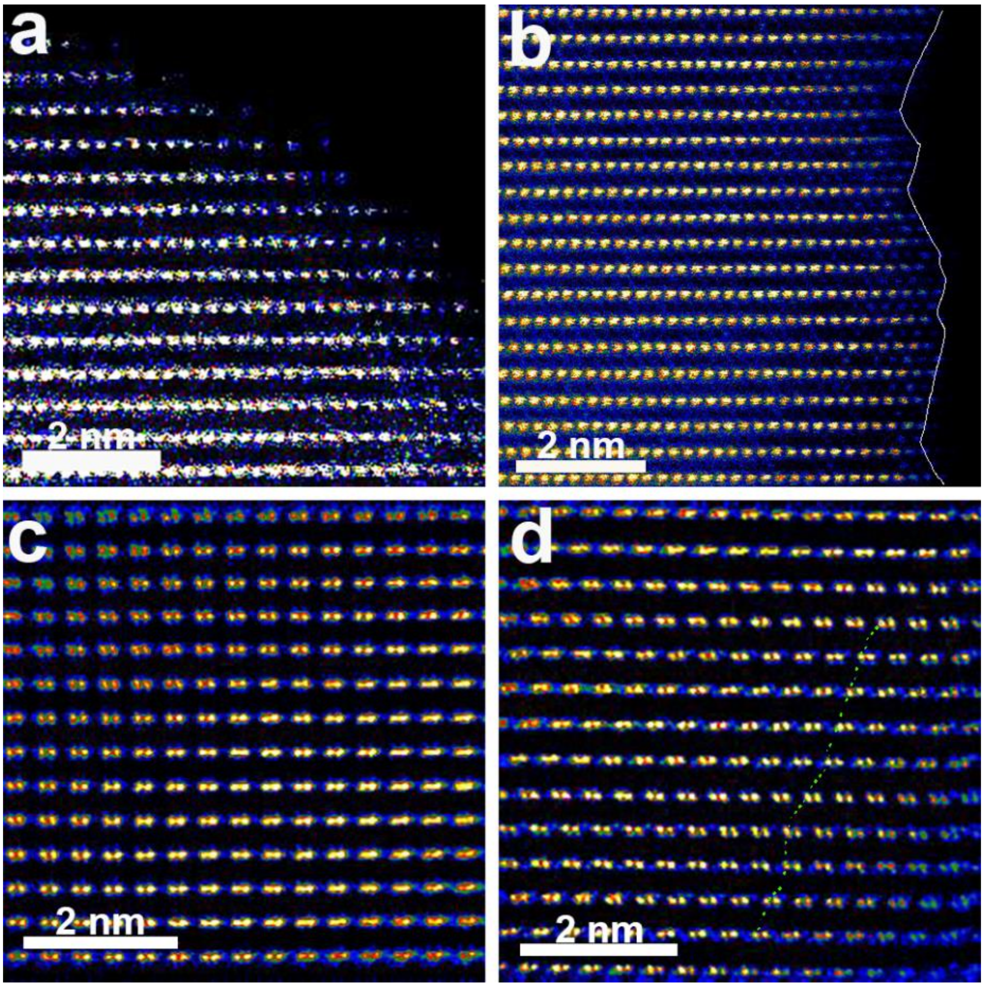

Supplementary Figure 2. Aberration-corrected scanning transmission electron microscopy images of the pristine (a and c) and M-LRO (b and d) samples. The boundary of the pristine sample is smoother than that of the M-LRO sample. In addition, there are some dislocations in the subsurface of the M-LRO sample.

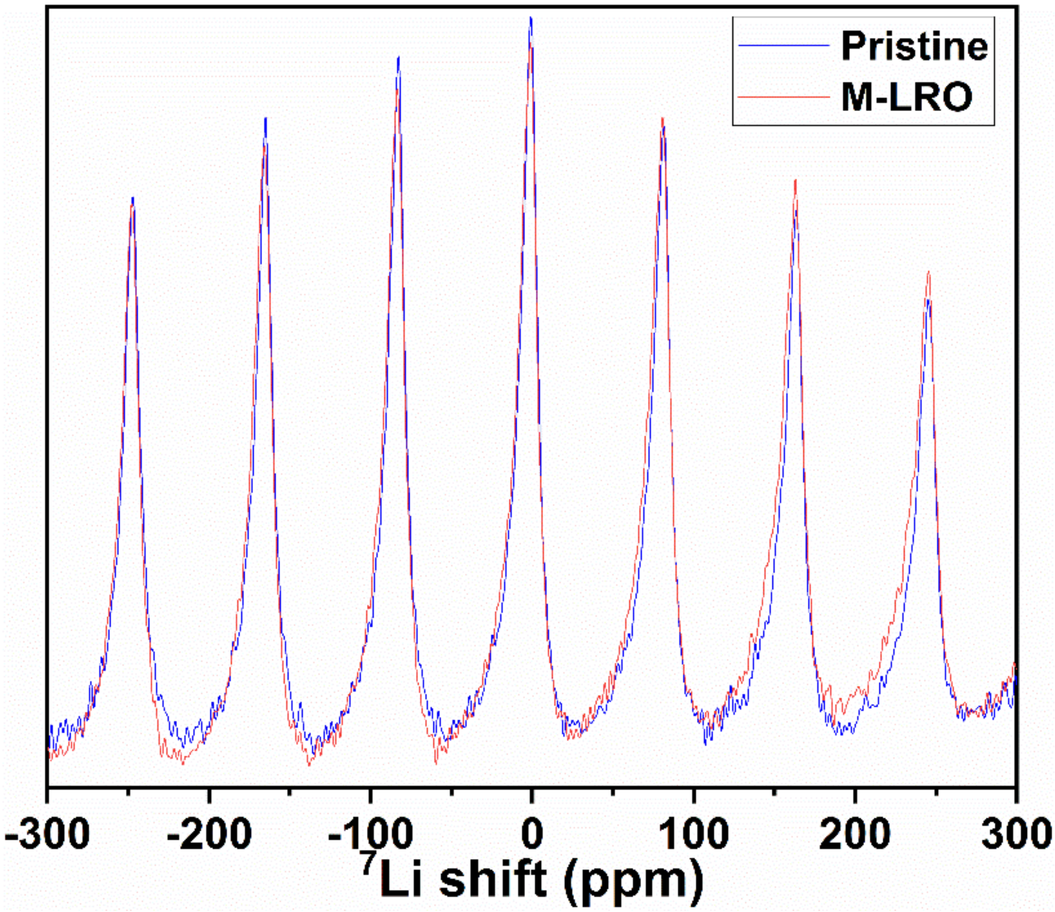

Supplementary Figure 3. ^{7}Li solid-state NMR spectra of both samples. The wider peaks indicate the existence of oxygen vacancies.

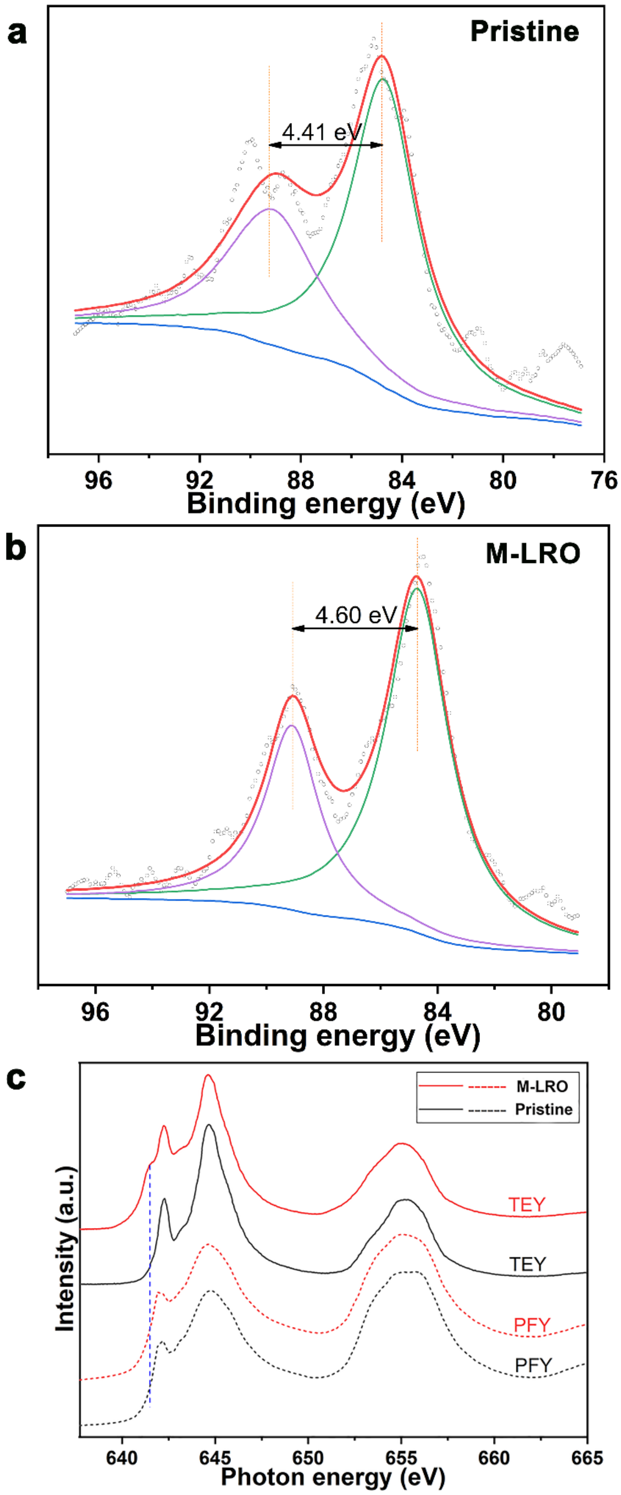

Supplementary Figure 4. Mn 3*s* XPS spectra of the pristine (a) and M-LRO samples (b) and Mn *L*-edge spectra of both samples in TEY and FY modes. According to the linear equation due to the relation between the splitting energy of the Mn 3*s* (ΔE_{3s}) level and Mn valence (ν_{Mn}):[1]

$$\nu_{Mn} = 9.67 - 1.27\Delta E_{3s} \,/\, \text{eV}.$$

The valence of Mn is 3.83 for the M-LRO sample, which means that the oxygen vacancy concentration is 5%. This agrees with the neutron powder diffraction refinement result. The Mn *L*-edge spectra illustrate that reduced Mn only exists on the surface of the M-LRO sample. The term a.u. means arbitrary units.

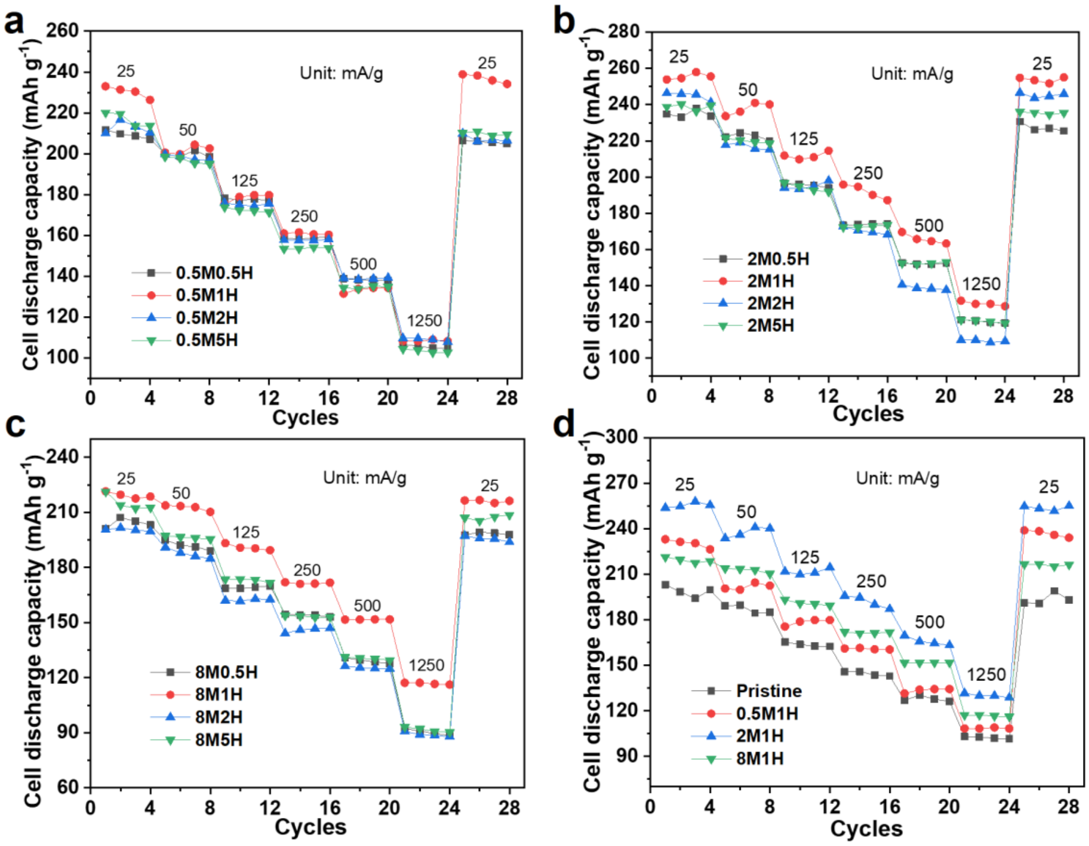

Supplementary Figure 5. Rate capacity of samples treated with different hydrazine concentrations and times. Specifically, the samples treated for 1 hour had the best performance whether in 0.5M, 2M or 8M hydrazine hydrate solution. In addition, the electrochemical performance of the samples treated with the three concentrations decreased as a whole with time over or less 1 hour. Therefore, too high of a concentration and too long of a processing time will increase the concentration of oxygen vacancies and reduction of TMs, hence making the crystal structure unstable. And too low concentrations and too short treatment time are not sufficient to modify the material, so the electrochemical performance is not improved significantly.

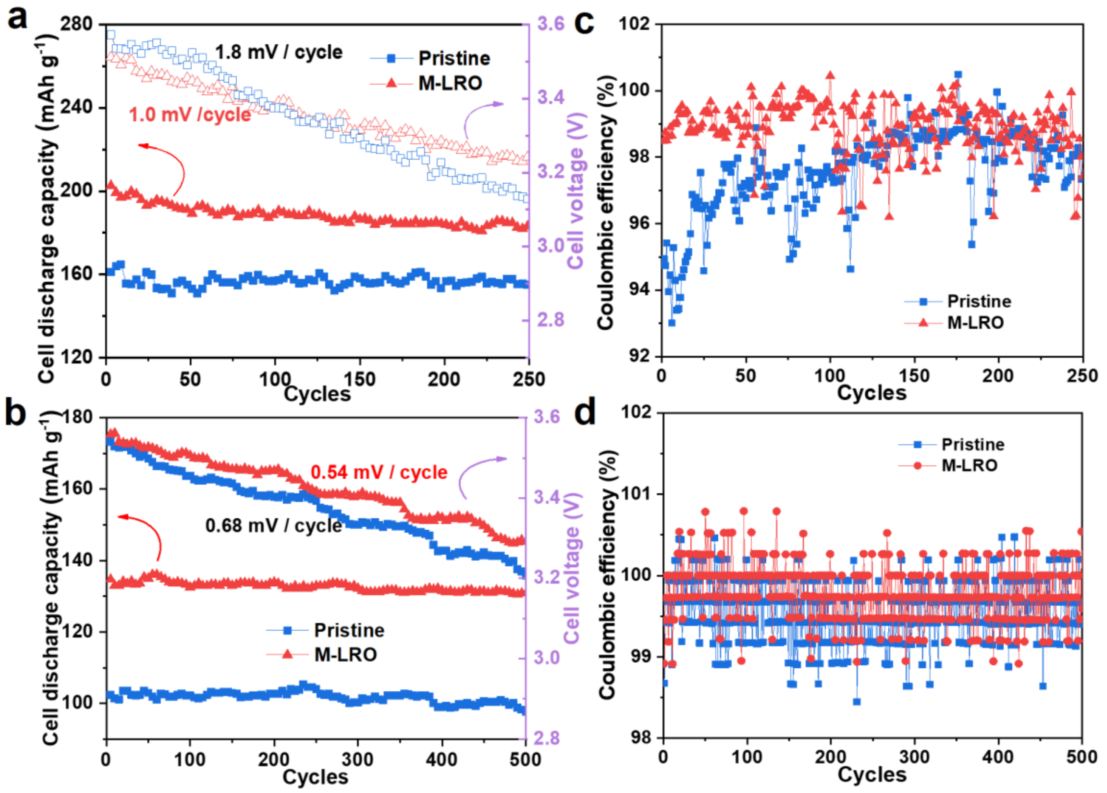

Supplementary Figure 6. (a and b) Cycling performance and voltage decay of both samples at specific currents of 250 mA/g and 1250 mA/g, respectively. (c and d) Coulombic efficiency of both samples at specific currents of 250 and 1250 mA/g, respectively.

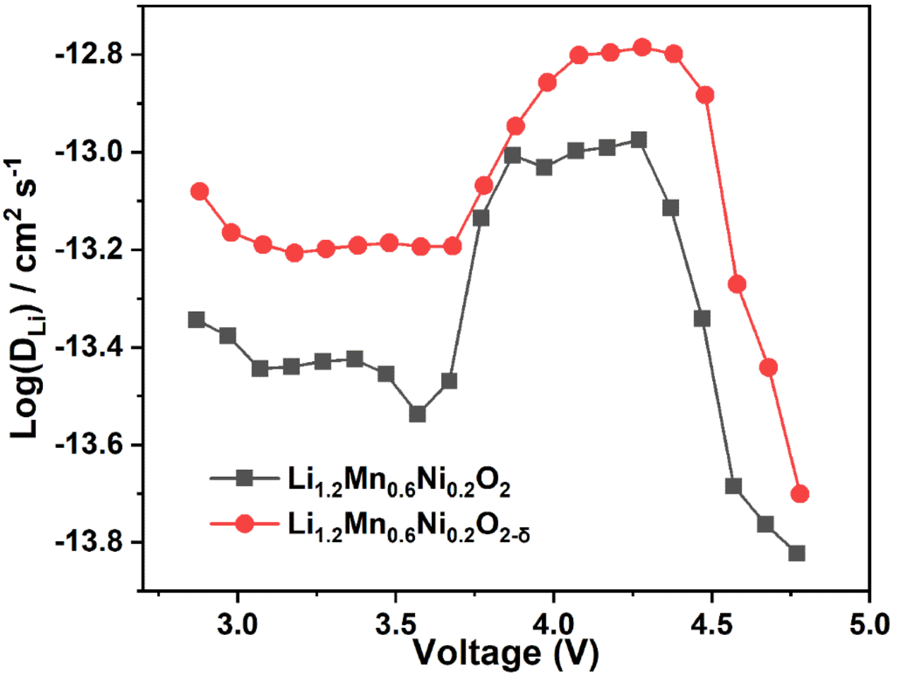

Supplementary Figure 7. PITT graph of both samples during the charging process. The diffusion coefficient (D_{Li^+}) can be calculated by the following equation:[2] $D_{Li^+} = -[\mathrm{d}\ \ln(I)/\mathrm{d}t]\cdot[4L^2/\pi^2]$, in which I and t stand for the titration current and time of each step, respectively, and L represents the diffusion length.

The D_{Li}^+ value of both samples decreases from the OCV to approximately 3.7 V during the first charging process, which may be caused by the narrowing of the Li^+ diffusion channel and the increased impedance with initial Li-ion extraction; this is consistent with the decreased c and a parameters according to previous papers.[3,4] From 3.8-4.5 V, an increase in the electrostatic repulsion between oxygen atoms occurs along with the further deintercalation of Li^+, which is beneficial to Li-ion diffusion due to the expanded I_{LiO2} layers. Additionally, because of the inherently larger I_{LiO2} layers in the modified sample, the Li^+ diffusion coefficient of the $Li_{1.2}Mn_{0.6}Ni_{0.2}O_{2-\delta}$ sample is better than that of the pristine sample. The decreased diffusion coefficient can be ascribed to the migration of additional nickel ions, the structural rearrangement that occurs with oxygen release, and the production of byproducts from 4.6-4.8 V, which also means that the kinetics of the cationic redox chemistry before 4.5 V are faster than those of the anionic redox chemistry after the voltage plateau. Specifically, the kinetics of oxygen redox are related to charge-transfer kinetics, and it stems from the distribution of the charge carrier Li^+/O^{n-} not being similar to free molecule diffusion, which can be simply described by Fick's law. Both the oxygen redox and continuous Li^+ migration will cause the local charge to be nonneutral. To maintain local charge neutrality, charge redistribution/transfer should occur when Li^+ and O^{n-} are diffused or redistributed. Therefore, the chemical Li^+ diffusion coefficient determined here is also coupled with a slow electron-transfer property that is sensitive to the oxygen redox process; this is why there is a significant decrease in the coefficient after 4.5 V.

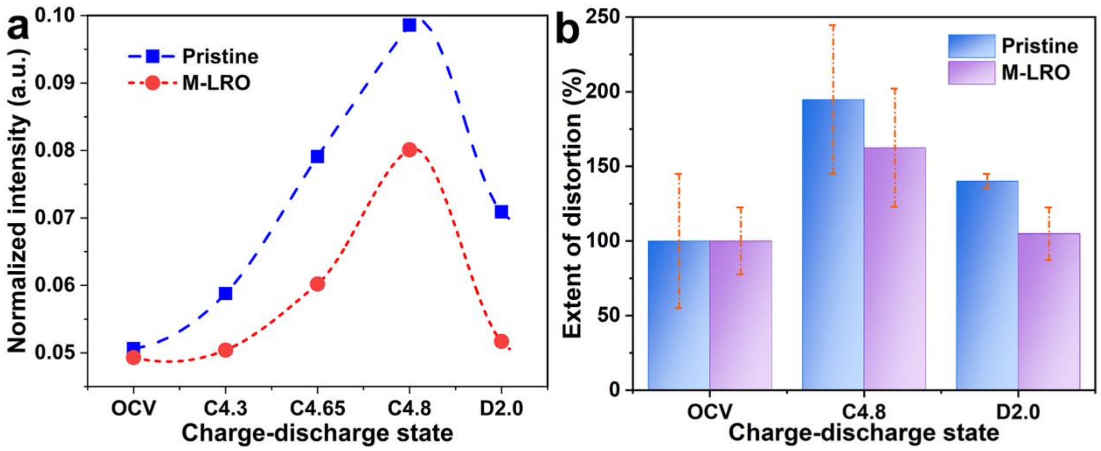

Supplementary Figure 8. Intensity changes of the Mn *K*-edge pre-edge peaks at different delithiation states (a). Relationship between the intensity and distortion extent is based on an OCV of 100%. The error bars indicate the standard deviation. The term a.u. means arbitrary units.

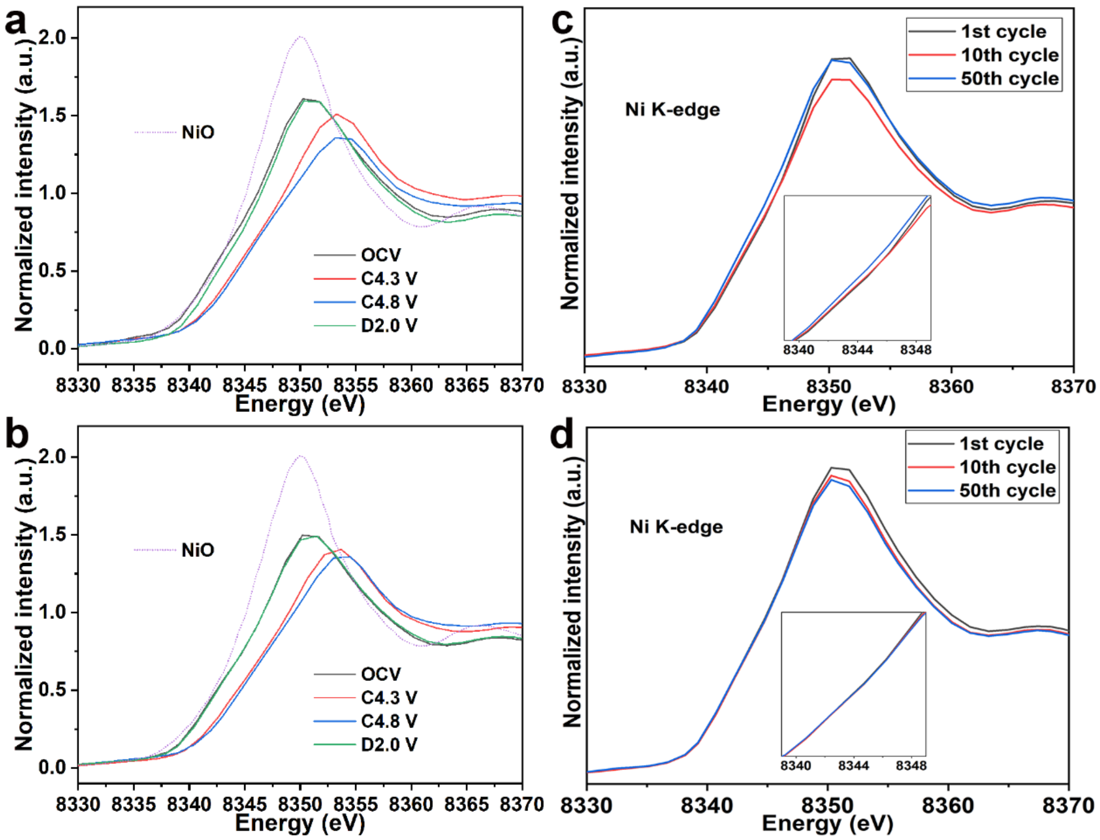

Supplementary Figure 9. *Ex situ* Ni *K*-edge spectra of both samples at different delithiation states and different cycles. (a and c) Pristine sample and (b and d) M-LRO sample. The spectrum of the M-LRO sample can return to the OCV state when discharged to 2.0 V in contrast to that of the pristine sample. In particular, the spectra of the M-LRO sample is nearly unchanged during the first 50 cycles. The term a.u. means arbitrary units.

Supplementary figure 9a and b show the *ex situ* Ni *K*-edge XAS spectra of the initial cycle of both samples. First, compared with NiO, the absorption edges of both samples do not completely overlap with that of NiO due to the difference in the coordination structure; however, the oxidation state of Ni shows a +2 valence. Then, with the gradual increase in voltage, the absorption edge of the Ni *K*-edge also gradually moves towards the higher energy direction, corresponding to the oxidation of Ni^{2+} to Ni^{4+}. Note that the absorption edges are similar when charged to 4.3 V and to 4.8 V, indicating that the charge compensation of Ni mainly occurs before the 4.5 V voltage plateau in the layered Li-rich oxides. However, the difference between the white line peaks of the pristine sample $Li_{1.2}Mn_{0.6}Ni_{0.2}O_2$ when charged to 4.3 V and 4.8 V is more significant than that of the modified sample $Li_{1.2}Mn_{0.6}Ni_{0.2}O_{2-\delta}$, which means that the local structure of Ni has changed during the oxidation process. This may be caused by the migration of Ni from the transition metal layer to the Li-ion layer. In addition, when discharged to 2.0 V, the absorption edge of the pristine sample cannot coincide with the OCV state, while the edge of the modified sample can perfectly overlap, which indicates that Ni irreversibly migrates from the transition metal layer to the Li-ion layer and is trapped in it during the charging-discharging process of the pristine sample. In comparison, Ni

is confined in the form of distorted octahedral NiO_6 (only a weak white line peak change) due to the softening of the structure of the sample with oxygen vacancies, thereby resulting in the complete recovery of the structure when discharged to 2.0 V.

Supplementary figure 9c and d show the Ni *K*-edge XAS results of $Li_{1.2}Mn_{0.6}Ni_{0.2}O_2$ and $Li_{1.2}Mn_{0.6}Ni_{0.2}O_{2-\delta}$ at different cycles, respectively. From the first cycle of discharging to 2.0 V to the 10th cycle and then to the 50th cycle, the absorption edge and white line peak of pristine $Li_{1.2}Mn_{0.6}Ni_{0.2}O_2$ gradually change, indicating that the electronic state and local structure of Ni change in the pristine sample. The modified sample hardly changes, meaning Ni is stable in the transition metal layer of $Li_{1.2}Mn_{0.6}Ni_{0.2}O_{2-\delta}$. In summary, oxygen vacancies and reduced Mn soften the crystal structure, which binds Ni to the transition metal layer, stabilizes the crystal structure, and improves the performance of the material.

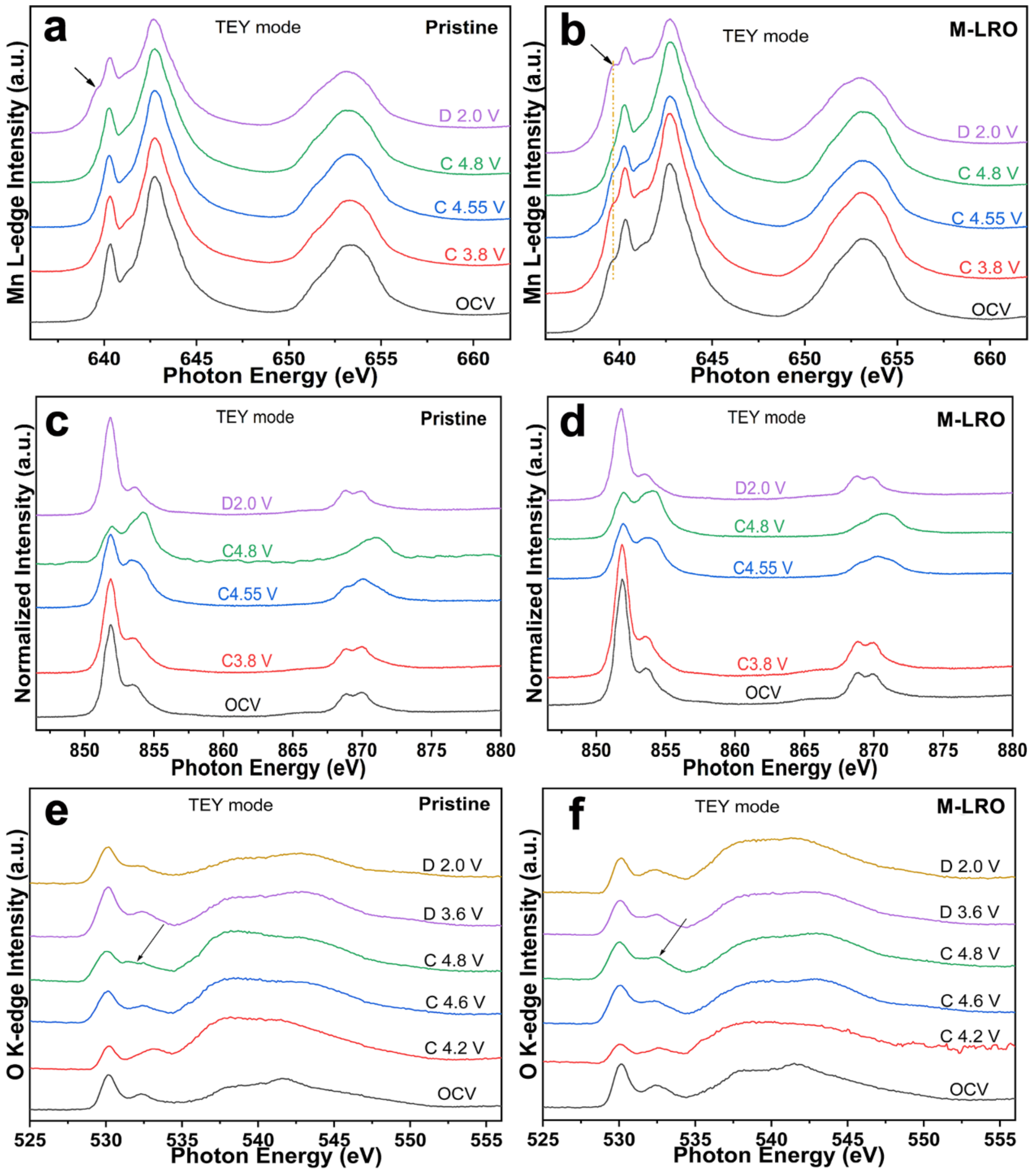

Supplementary Figure 10. *Ex situ* soft XAS of both samples in TEY mode. (a and b) Mn *L*-edge spectra of the pristine and M-LRO samples. (c and d) Ni *L*-edge spectra of the pristine and M-LRO samples. (e and f) O *K*-edge spectra of the pristine and M-LRO samples. The arrows represent Mn^{3+} in Figure a and b. In Figure e and f, the arrows indicate the e_g orbitals. The term a.u. means arbitrary units.

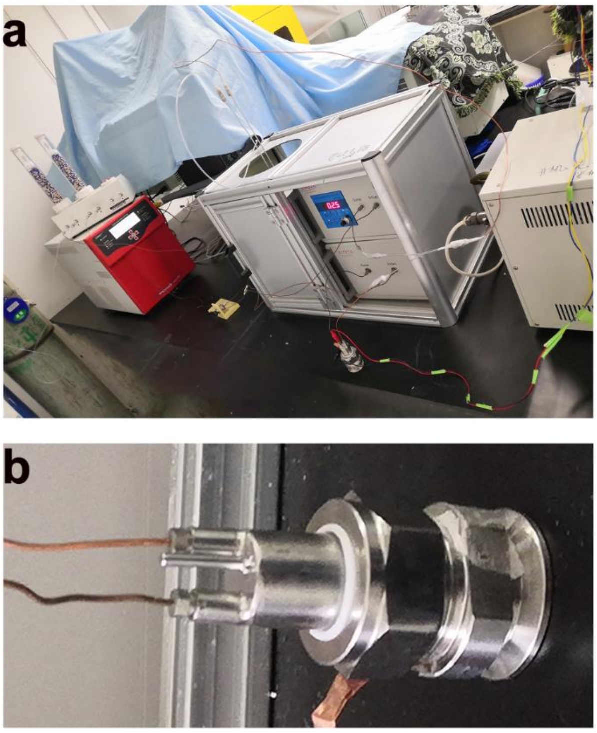

Supplementary Figure 11. (a) Image of the operando DEMS instrument. (b) Close-up of the DEMS Swagelok cell.

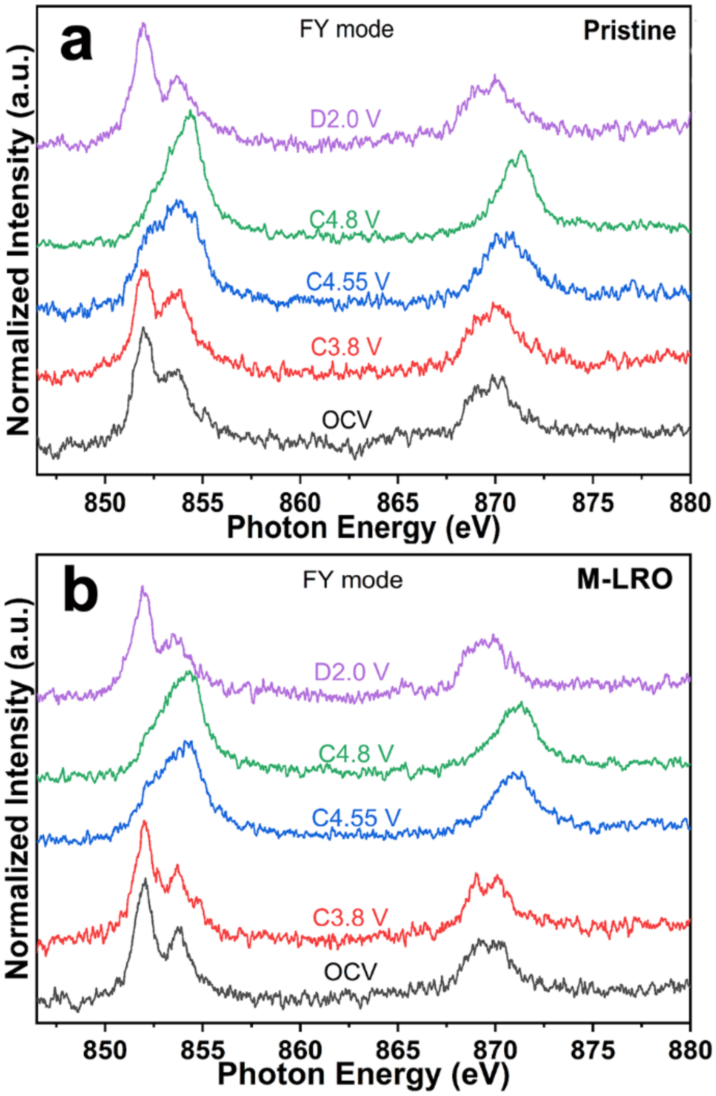

Supplementary Figure 12. *Ex situ* Ni *L*-edge XAS spectra of both samples in FY mode at different charge–discharge states. The term a.u. means arbitrary units.

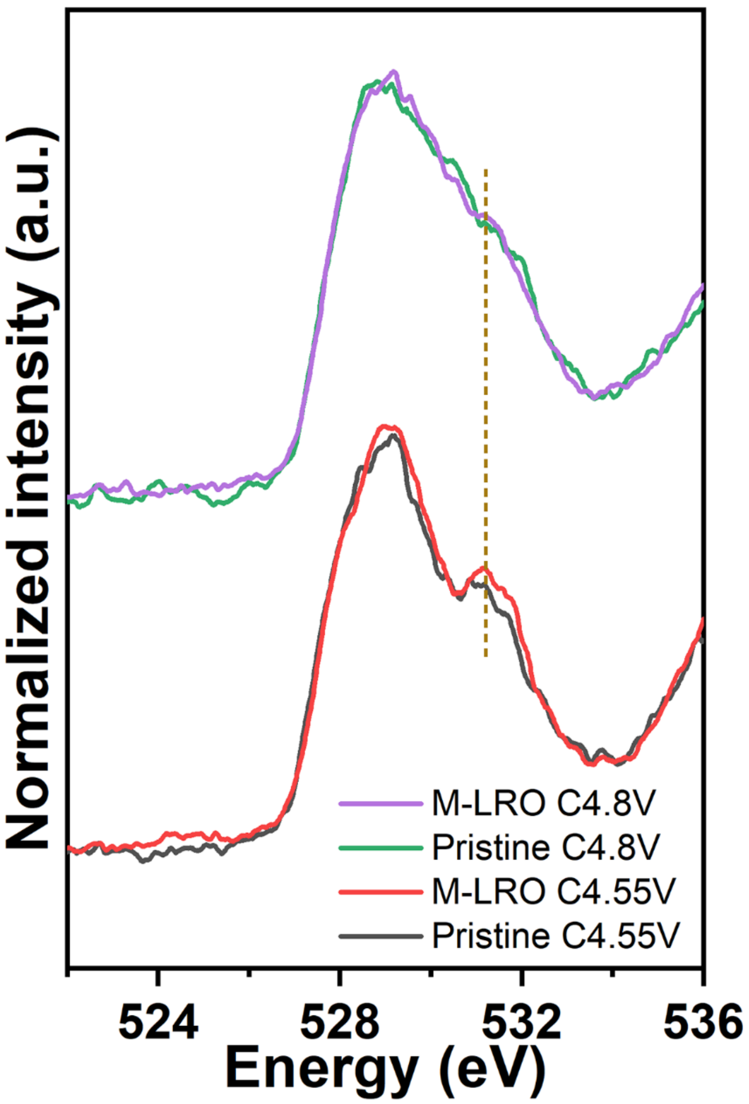

Supplementary Figure 13. *Ex situ* soft XAS of both samples when charged to 4.55 V and to 4.80 V. The term a.u. means arbitrary units.

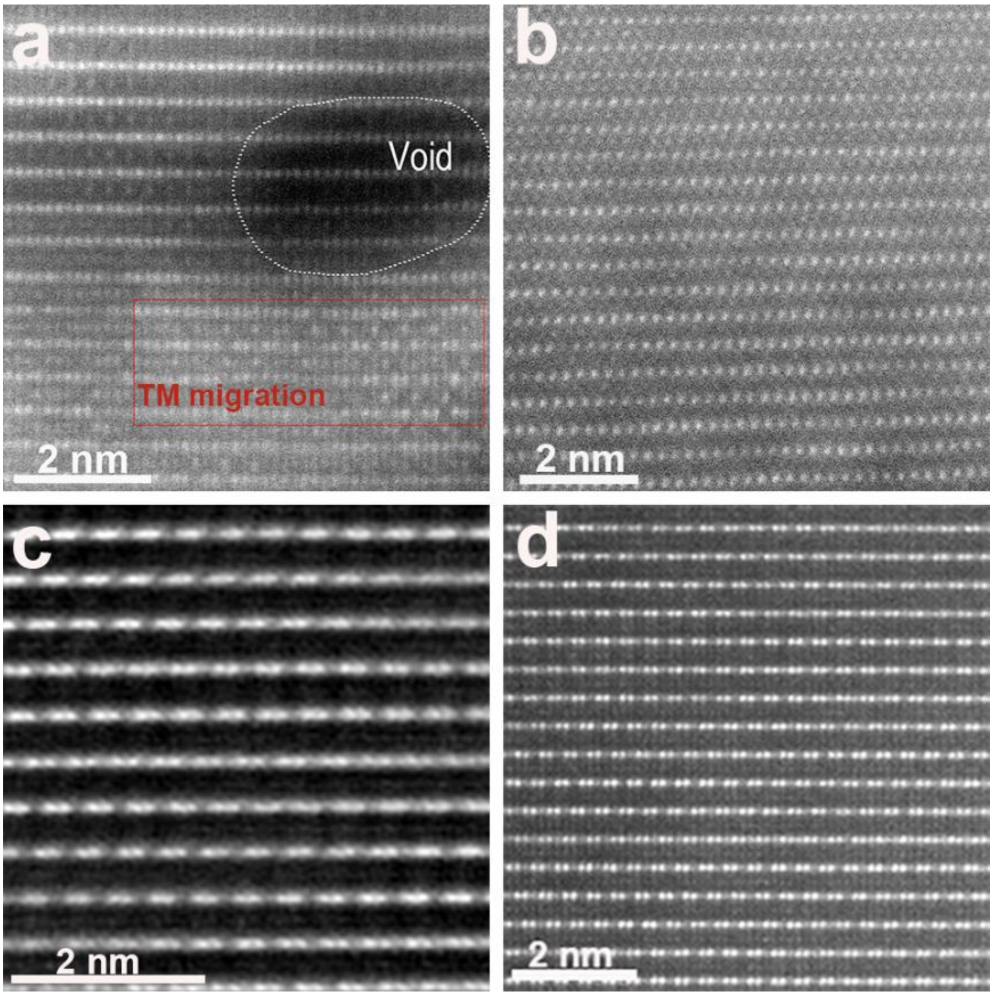

Supplementary Figure 14. *Ex situ* HAADF-STEM images of the pristine (a) and M-LRO (b) samples charged to 4.8 V. HAADF-STEM images of the pristine (c) and M-LRO (d) samples discharged to 2.0 V. The bright spots represent Ni and Mn, which have higher atomic numbers than the other elements. Therefore, there is serious TM migration in the pristine sample.

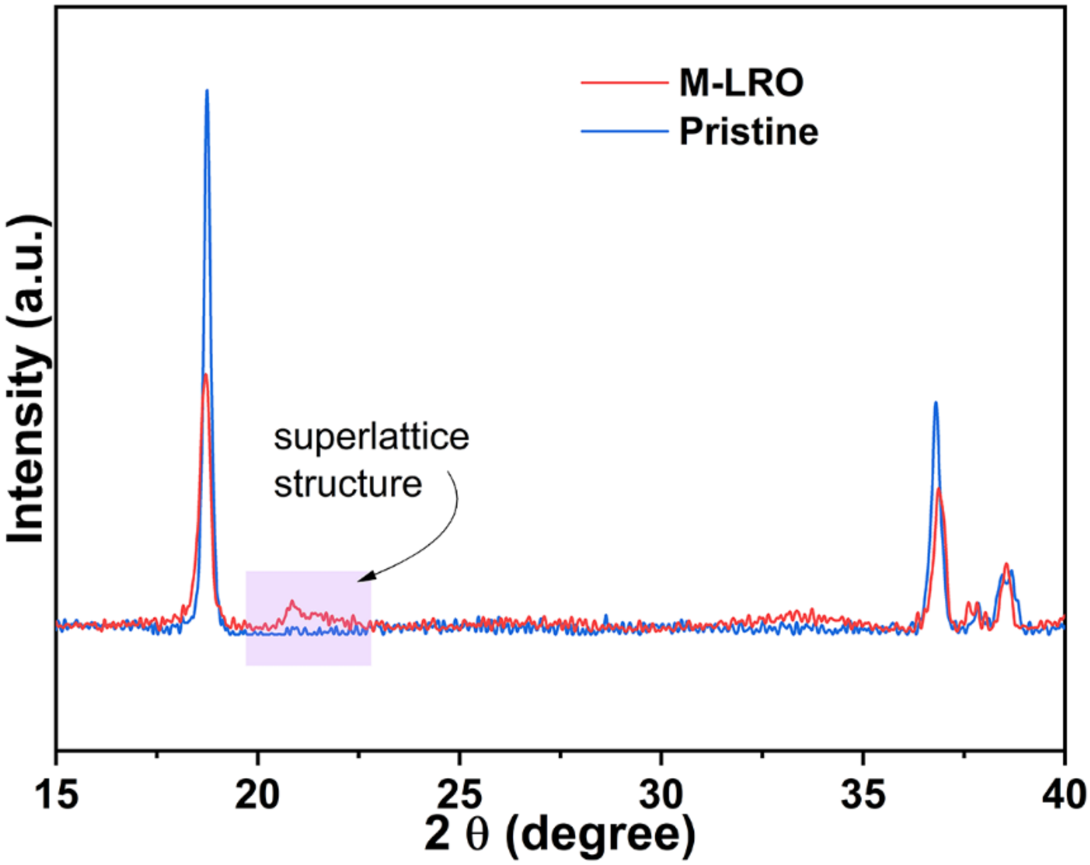

Supplementary Figure 15. *Ex situ* XRD of both samples when discharged to 2.0 V. The area shaded in purple shows the superlattice structure. The loss of the superstructure peaks for the pristine sample means that the TMs irreversibly migrate from the broken honeycomb ordering. The term a.u. means arbitrary units.

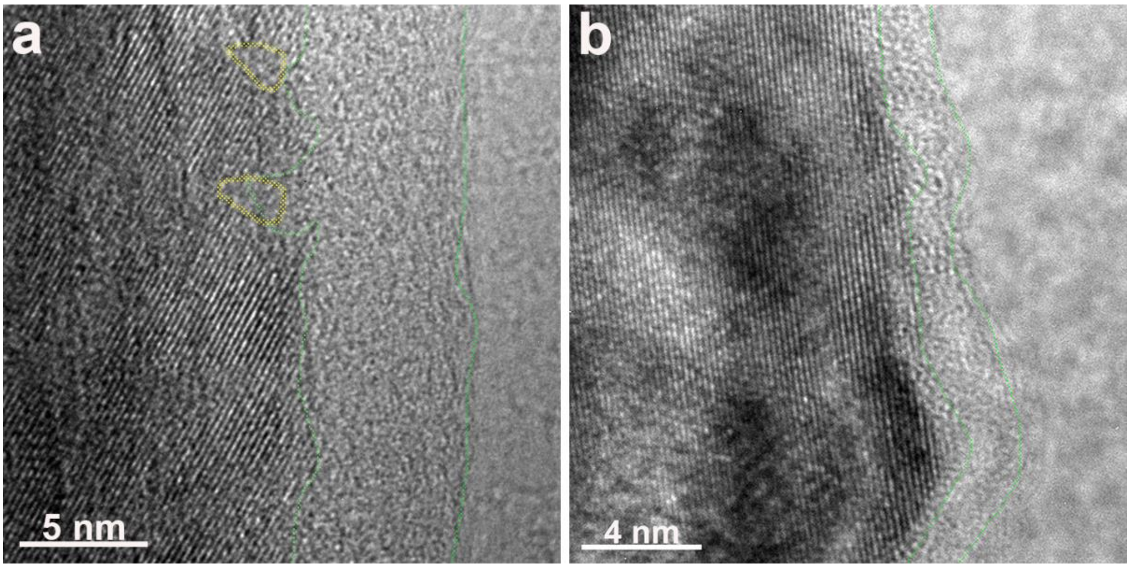

Supplementary Figure 16. *Ex situ* high-resolution TEM images of (the a) pristine and (b) M-LRO samples after 100 cycles at a current density of 250 mA/g.

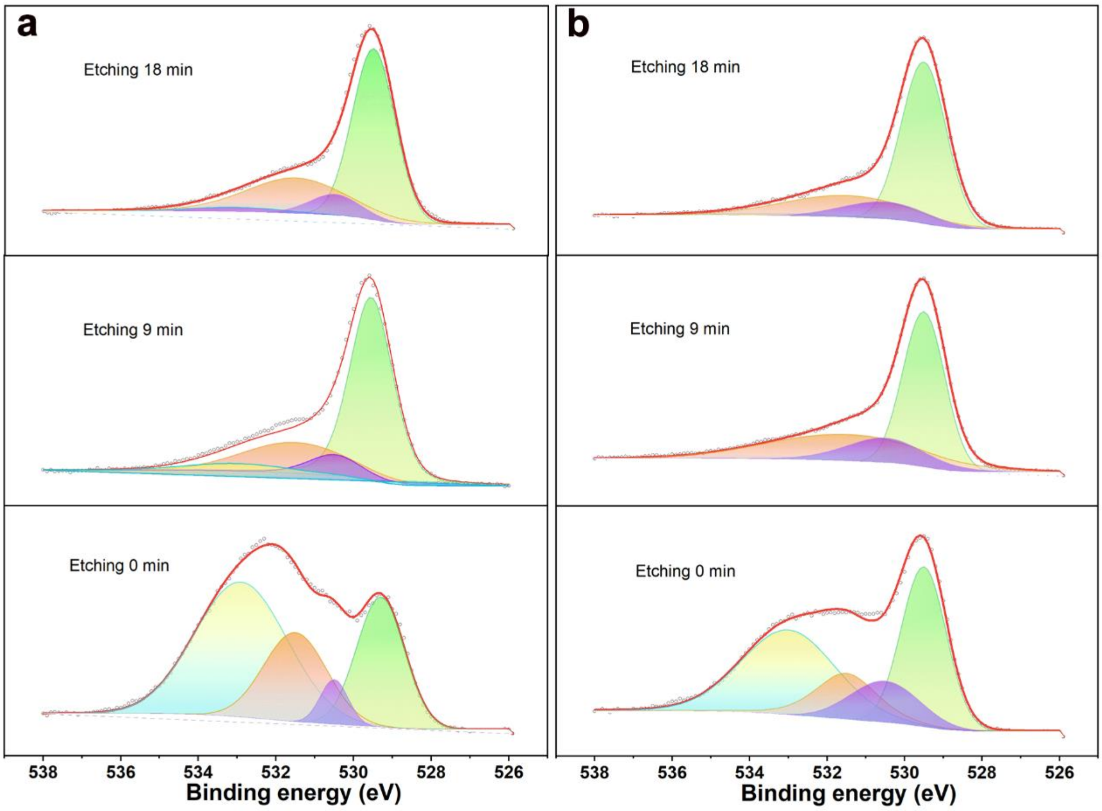

Supplementary Figure 17. *Ex situ* O 1*s* XPS of both samples after different etching times. (a) Pristine sample charged to 4.8 V and (b) M-LRO sample charged to4.8 V. The light blue area (533 eV) indicates the oxidation species of the electrolyte, the saffron region (531.5 eV) represents the surface absorbed species, the purple area (530.5 eV) indicates the lattice oxygen (O^{-}/O_2^{2-}), and the green area (529.5 eV) indicates the crystal O^{2-} network.

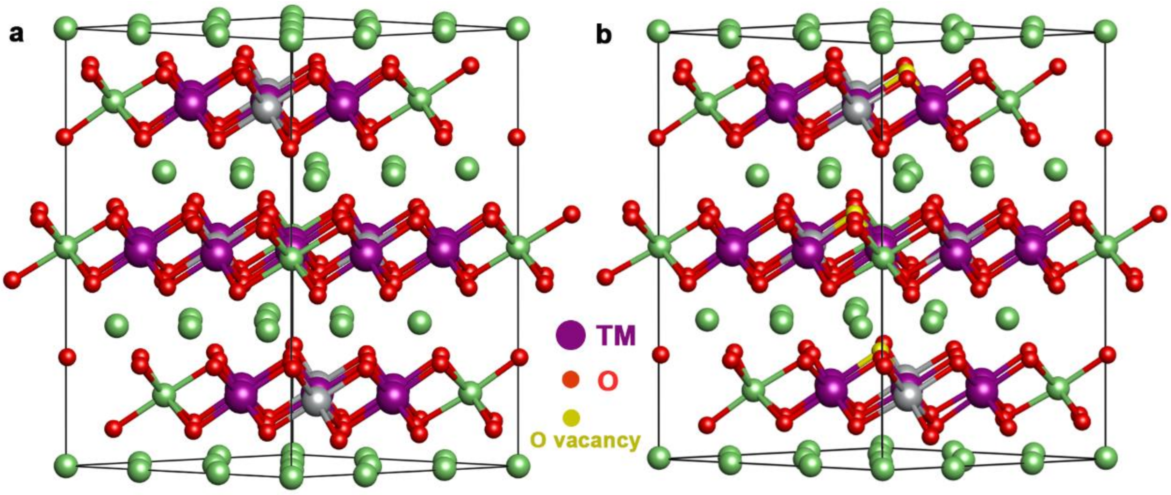

Supplementary Figure 18. DFT calculation models: (a) pristine sample and (b) M-LRO sample.

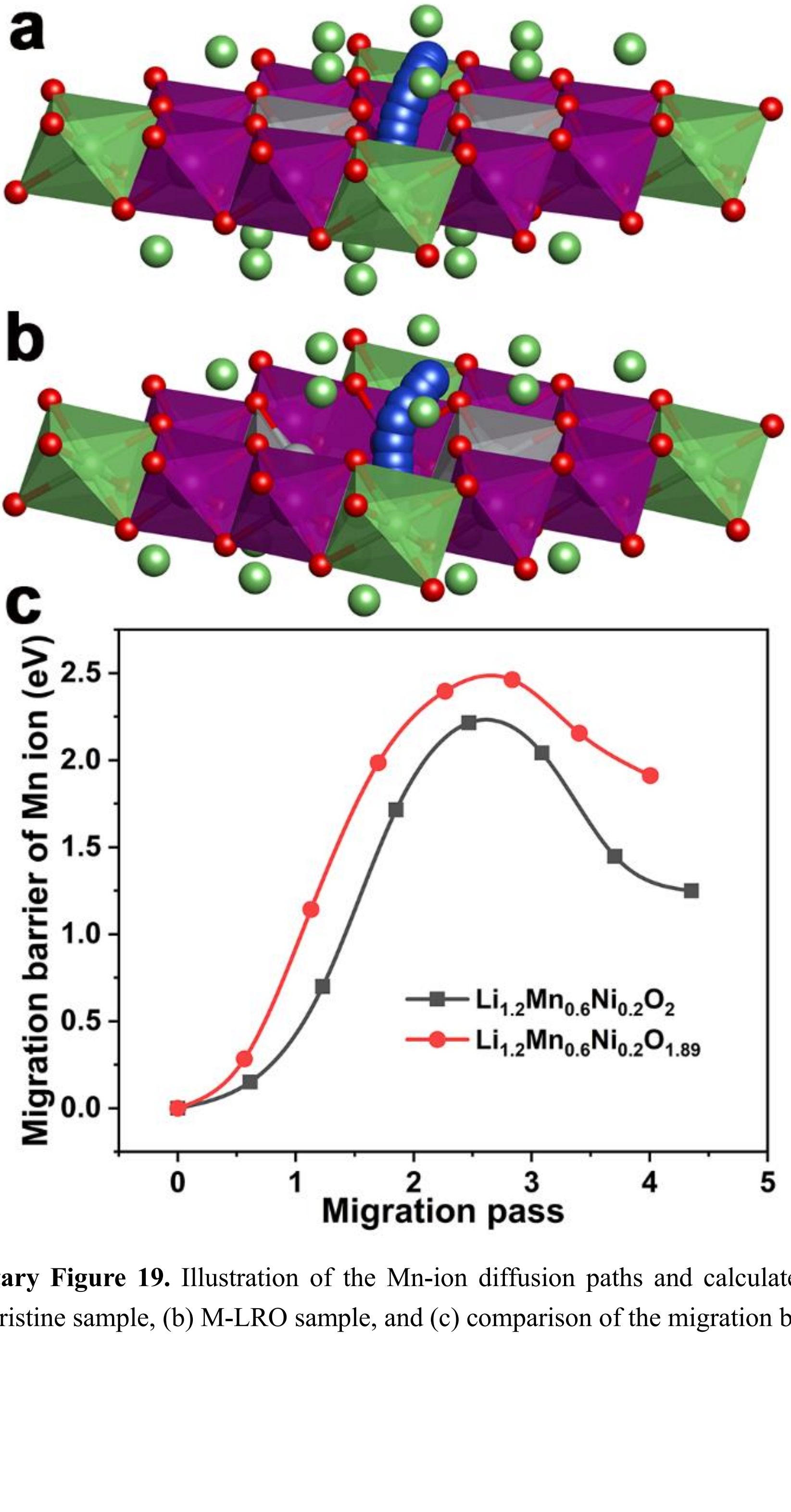

Supplementary Figure 19. Illustration of the Mn-ion diffusion paths and calculated migration barrier: (a) pristine sample, (b) M-LRO sample, and (c) comparison of the migration barrier.

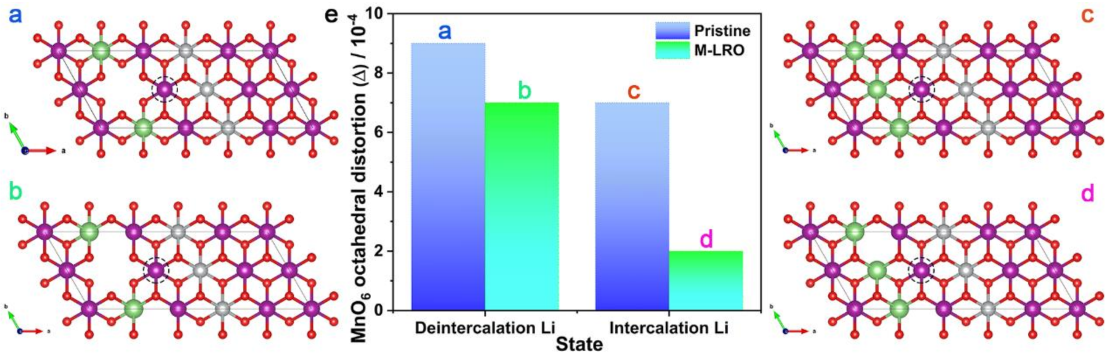

Supplementary Figure 20. Octahedral MnO_6 distortion (Δ). The calculation formula is $\Delta = \frac{1}{6}\sum(\frac{R_i - R_{av}}{R_{av}})^2$, where R_{av} represents the average bond length and R_i represents the individual length of the Mn-O bond in the octahedral MnO_6 structure.[5]

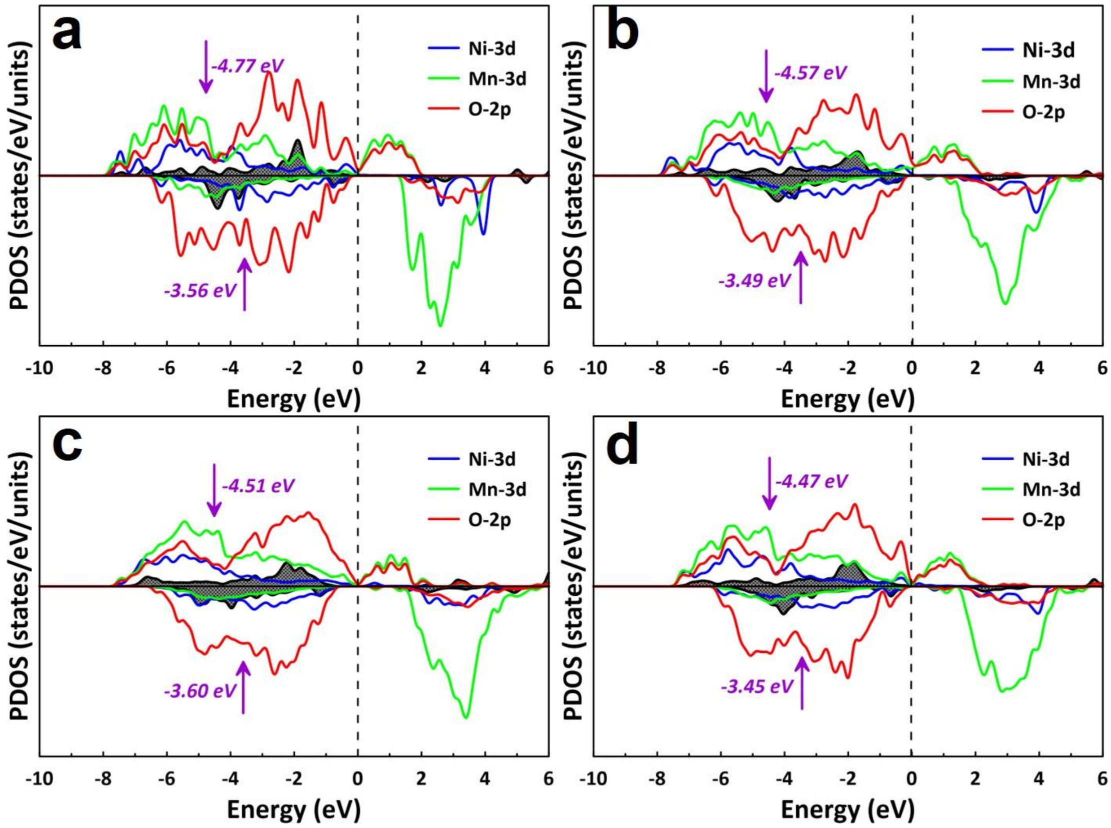

Supplementary Figure 21. DOS patterns of both samples at different charge–discharge states. Initial states of the pristine (a) and M-LRO (b) samples. Relithiation state after the delithiation of pristine (c) and M-LRO (d). The shaded area indicates the Li-O-Li state. From the patterns, the d band centre states of the M-LRO sample are closer to the Fermi level, which illustrates that Mott-Hubbard splitting is narrower and that U is smaller. When Li^+ is reinserted into the layered structure, the splitting gap contracts for both samples; however, the M-LRO sample more easily returns to its initial state. These calculation results are in agreement with the Mn L-edge RIXS spectra.

Supplementary Table 1. Refinement of the neutron diffraction results of both samples

Sample	a (Å)	c (Å)	V (Å ^{3})	Z_{ox}	$S_{(MO2)}$ (Å)	$I_{(LiO2)}$ (Å)	TM-O (Å)	R_p (%)
Pristine	2.8573(4)	14.2527(3)	100.77(3)	0.24095	2.6334	2.1175	1.9603(4)	4.51
M-LRO	2.8614(9)	14.2612(5)	101.128(5)	0.24162	2.6246	2.1316	1.9660(4)	5.31

Supplementary Table 2. Atomic occupancy information of both samples from the NPD Rietveld refinement

Sample	Atom	Site	x	y	z	Occupation
Pristine	Li1	3a	0	0	0	0.981(5)
	Ni1	3a	0	0	0	0.018(5)
	Mn	3b	0	0	0.5	0.600
	Ni2	3b	0	0	0.5	0.181(5)
	Li2	3b	0	0	0.5	0.218(6)
	O	6c	0	0	0.24095(5)	1.000(4)
M-LRO	Li1	3a	0	0	0	0.959(2)
	Ni1	3a	0	0	0	0.040(8)
	Mn	3b	0	0	0.5	0.600
	Ni2	3b	0	0	0.5	0.159(2)
	Li2	3b	0	0	0.5	0.240(8)
	O	6c	0	0	0.24136(6)	0.950(6)

Reference

1. Beyreuther, E., Grafström, S., Eng, L. M., Thiele, C. & Dörr, K. XPS investigation of Mn valence in lanthanum manganite thin films under variation of oxygen content. *Phys. Rev. B* **73**, 155425 (2006).

2. Li, Q., Zhou, D., Zhang, L., Ning, D., Chen, Z., Xu, Z., Gao, R., Liu, X., Xie, D., Schumacher, G. & Liu, X. Tuning Anionic Redox Activity and Reversibility for a High-Capacity Li-Rich Mn-Based Oxide Cathode via an Integrated Strategy. *Adv. Funct. Mater.* **29**, 1806706 (2019).

3. Shen, C. H., Huang, L., Lin, Z., Shen, S. Y., Wang, Q., Su, H., Fu, F. & Zheng, X. M. Kinetics and structural changes of Li-rich layered oxide $0.5Li_2MnO_3\cdot0.5LiNi_{0.292}Co_{0.375}Mn_{0.333}O_2$ material investigated by a novel technique combining in situ XRD and a multipotential step. *ACS Appl. Mater. Interfaces* **6**, 13271-13279 (2014).

4. Zhang, C., Liu, M., Pan, G., Liu, S., Liu, D., Chen, C., Su, J., Huang, T. & Yu, A. Enhanced Electrochemical Performance of $LiNi_{0.8}Co_{0.1}Mn_{0.1}O_2$ Cathode for Lithium-Ion Batteries by Precursor Preoxidation. *ACS Appl. Energy Mater.* **1**, 4374-4384 (2018).

5. Zhang, X., Pei, C., Chang, X., Chen, S., Liu, R., Zhao, Z. J., Mu, R. & Gong, J. FeO_6 Octahedral Distortion Activates Lattice Oxygen in Perovskite Ferrite for Methane Partial Oxidation Coupled with CO_2 Splitting. *J. Am. Chem. Soc.* **142**, 11540-11549 (2020).
